# Crystal structure of [1-(2,6-diiso­propyl­phen­yl)-2,4-bis­(di­methyl­amino)-5-tri­methyl­silyl-1,3,5-tri­aza­penta­dienyl-κ^2^ *N* ^1^,*N* ^5^](tri­phenyl­phosphane-κ*P*)copper(I)

**DOI:** 10.1107/S2056989015002169

**Published:** 2015-02-07

**Authors:** Feiguang Li, Lei Yan, Hongbo Tong, Meisu Zhou

**Affiliations:** a Institute of Applied Chemistry, Shanxi University, Taiyuan, Shanxi 030006, People’s Republic of China

**Keywords:** crystal structure, tri­aza­penta­dien­yl, copper(I) complex

## Abstract

The title complex, [Cu(C_21_H_38_N_5_Si)(C_18_H_15_P)], was obtained from the one-pot reaction between (Dipp)N(Li)SiMe_3_ (Dipp = 2,6-diiso­propyl­phen­yl), Me_2_NCN, CuCl and PPh_3_. The Cu^I^ atom has a distorted trigonal–planar coordination sphere. The tri­aza­penta­dienyl ligand acts as a κ^2^-donor. The N—Cu—N bond angle is 95.88 (14)°. In the tri­aza­penta­dienyl fragment, the C—N bond lengths are in the range 1.328 (5)–1.349 (5) Å, which indicates delocalization of the π-electrons in the NCNCN system.

## Related literature   

For reviews of related ligands and metals, see: Dias & Singh (2004 ▸); Flores *et al.* (2009 ▸); Xie *et al.* (2014 ▸); Zhou *et al.* (2008 ▸, 2011 ▸); Liu *et al.* (2013 ▸).
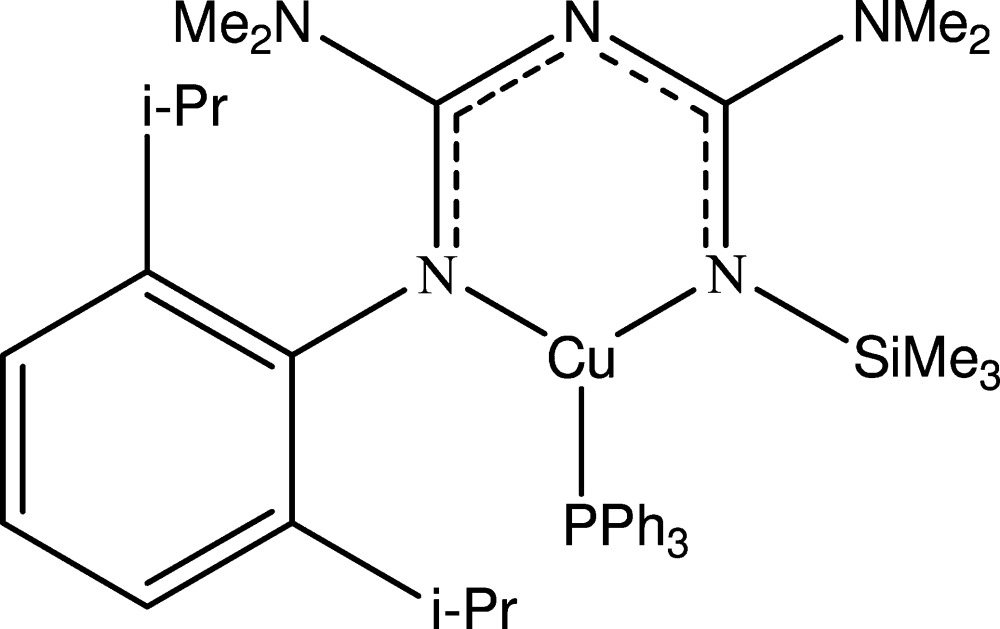


## Experimental   

### Crystal data  

[Cu(C_21_H_38_N_5_Si)(C_18_H_15_P)]
*M* *_r_* = 714.46
Triclinic, 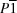

*a* = 9.7935 (16) Å
*b* = 11.2141 (18) Å
*c* = 19.570 (3) Å
α = 103.601 (4)°
β = 90.867 (3)°
γ = 108.240 (4)°
*V* = 1974.8 (6) Å^3^
*Z* = 2
Mo *K*α radiation
μ = 0.66 mm^−1^
*T* = 195 K
0.32 × 0.31 × 0.28 mm

### Data collection  

Bruker APEXII CCD diffractometer
Absorption correction: multi-scan (*SADABS*; Bruker, 2000 ▸) *T*_min_ = 0.818, *T*_max_ = 0.838
11084 measured reflections
6988 independent reflections
3246 reflections with *I* > 2σ(*I*)
*R*_int_ = 0.077

### Refinement  

*R*[*F* ^2^ > 2σ(*F* ^2^)] = 0.051
*wR*(*F* ^2^) = 0.089
*S* = 0.75
6988 reflections
435 parameters
12 restraints
H-atom parameters constrained
Δρ_max_ = 0.35 e Å^−3^
Δρ_min_ = −0.24 e Å^−3^

### 

Data collection: *APEX2* (Bruker, 2000 ▸); cell refinement: *SAINT* (Bruker, 2000 ▸); data reduction: *SAINT*; program(s) used to solve structure: *SHELXS97* (Sheldrick, 2008 ▸); program(s) used to refine structure: *SHELXL97* (Sheldrick, 2008 ▸); molecular graphics: *SHELXTL* (Sheldrick, 2008 ▸); software used to prepare material for publication: *SHELXTL*.

## Supplementary Material

Crystal structure: contains datablock(s) I, New_Global_Publ_Block. DOI: 10.1107/S2056989015002169/lx2294sup1.cif

Structure factors: contains datablock(s) I. DOI: 10.1107/S2056989015002169/lx2294Isup2.hkl

Supporting information file. DOI: 10.1107/S2056989015002169/lx2294Isup3.txt

**Fig. 1.**
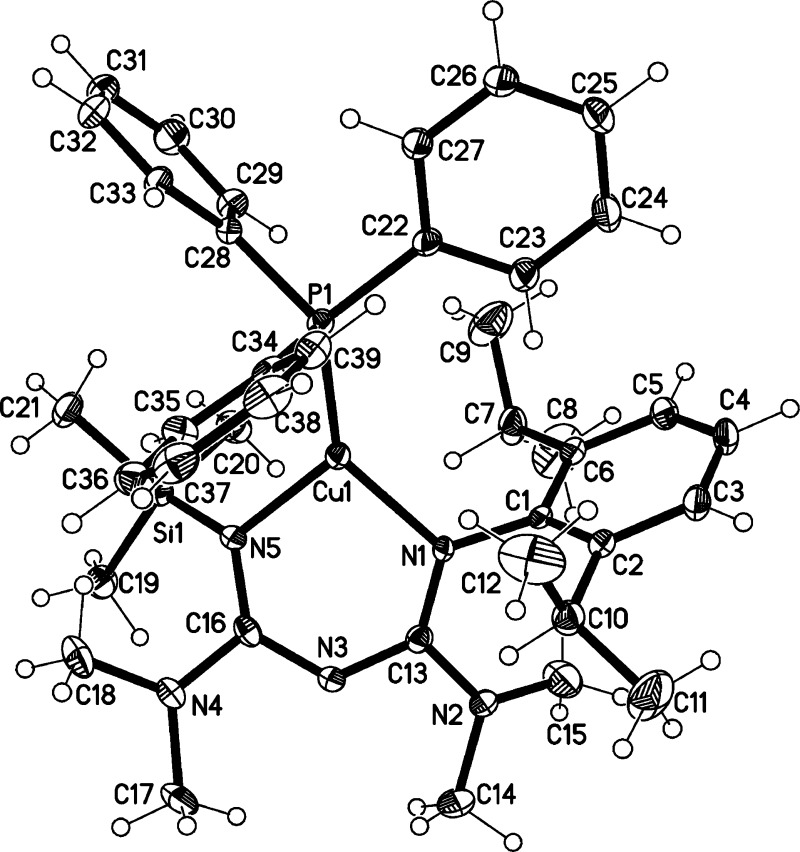
The molecular structure of (I), showing the atom-numbering scheme. Displacement ellipsoids are drawn at the 30% probability level. H atoms are presented as a small spheres of arbitrary radius.

. DOI: 10.1107/S2056989015002169/lx2294fig1.tif
The mol­ecular structure of (I), showing the atom-numbering scheme. Displacement ellipsoids are drawn at the 30% probability level. H atoms are presented as a small spheres of arbitrary radius.

CCDC reference: 1046645

Additional supporting information:  crystallographic information; 3D view; checkCIF report

## supplementary crystallographic information

### S1. Synthesis and crystallization

Me_2_NCN (0.41 mL, 5.06 mmol) was added to a solution of (Dipp)N(Li)SiMe_3_ (0.65 g, 2.53 mmol) in Et_2_O (30 cm^3^) at -78°C. The resulting mixture was warmed to *ca.* 25°C and stirred overnight. CuCl (0.25 g, 2.53 mmol) and PPh_3_ (0.66 g, 2.53 mmol) were added at -78°C. The resulting mixture was allowed to warm to *ca.* 25°C and stirred overnight. Filtered and the filtrate was concentrated in *vacuo* and stored at 25°C for 4 d, yielding colorless crystals of the title compound (0.62 g, 34 %).

Anal. calcd. for C_39_H_53_N_5_PSiCu (%): C, 65.56; H, 7.48; N, 9.80. Found: C, 65.59; H, 7.50; N, 9.76. All manipulations were performed under argon using standard Schlenk and vacuum line techniques. Et_2_O was dried and distilled over Na under argon prior to use. Elemental analysis is completely in agreement with the structure of the compound.

### S2. Refinement

All H atoms were positioned geometrically and refined using a riding model, with C—H = 0.95 Å for aryl, 1.00 Å for methine and 0.98 Å for methyl H atoms, respectively. *U*_iso_(H) = 1.2*U*_eq_(C) for aryl and methine, and 1.5*U*_eq_(C) for methyl H atoms. The positions of methyl hydrogens were optimized using the SHELXL-97's command AFIX 137 (Sheldrick, 2008).

### S3. Results and discussion

1,3,5–Tri­aza­penta­dienyl ligands are one of the most useful nitro­gen-based ligands in coordination chemistry and organometallic chemistry due to their structural features and stronger coordination properties(Dias & Singh, 2004; Flores *et al.*, 2009). Our group has developed a series of 2,4–N,N'–substituted–1,3,5–tri­aza­penta­dienyl ligands and obtained their Fe,Co,Mg,Cu(I) complexes (Xie *et al.* , 2014; Zhou *et al.*, 2011; Liu *et al.*, 2013).The title compound is polymorph of C_39_H_53_N_5_PSiCu which was reported in 2008(Zhou *et al.*, 2008).This article will provide a new set of unit cell data on the 1,3,5– tri­aza­penta­dienyl (tri­phenyl­phosphane) copper(I) compound C_39_H_53_N_5_PSiCu.

Its molecular structure is shown in Fig. 1. In the monomeric molecular structure of title compound, the dihedral angle between N1C13C16N3 and C13N2C16 is 14.16(0.65)°, and the dihedral angle between N1C13C16N3 and N1CuN3 is 3.30(0.33)°. The bond angles of N5—C16—N3, C16—N3—C13, and N3—C13—N1 are 127.8 (4), 125.8 (4), and 124.6 (4)°, respectively. The N1C13C16N3 plane is twisted by about 26.47(0.28)° from the N1N5CuP plane. The tri­aza­penta­dienyl ligand acts as a κ^2^-donor. The bond angle of N1—Cu—N5 is 95.88 (14)°. In the tri­aza­penta­dienyl fragment, the C—N bond distances are in the range of 1.328 (5)–1.349 (5) Å, which indicates the delocalization of the π-electrons as a η^5^ anion in the NCNCN system.Cu located above 0.172 (2) Å from P1–N1–N5 triangular plane.

### Crystal data

**Table:**

[Cu(C_21_H_38_N_5_Si)(C_18_H_15_P)]	*Z* = 2
*M**_r_* = 714.46	*F*(000) = 760
Triclinic, *P*1	*D*_x_ = 1.202 Mg m^−^^3^
Hall symbol: -P 1	Mo *K*α radiation, λ = 0.71073 Å
*a* = 9.7935 (16) Å	Cell parameters from 1276 reflections
*b* = 11.2141 (18) Å	θ = 2.5–18.7°
*c* = 19.570 (3) Å	µ = 0.66 mm^−^^1^
α = 103.601 (4)°	*T* = 195 K
β = 90.867 (3)°	Block, colorless
γ = 108.240 (4)°	0.32 × 0.31 × 0.28 mm
*V* = 1974.8 (6) Å^3^	

### Data collection

**Table:**

Bruker APEXII CCD diffractometer	6988 independent reflections
Radiation source: fine-focus sealed tube	3246 reflections with *I* > 2σ(*I*)
Graphite monochromator	*R*_int_ = 0.077
φ and ω scans	θ_max_ = 25.1°, θ_min_ = 1.1°
Absorption correction: multi-scan (*SADABS*; Bruker, 2000)	*h* = −11→9
*T*_min_ = 0.818, *T*_max_ = 0.838	*k* = −11→13
11084 measured reflections	*l* = −23→23

### Refinement

**Table:**

Refinement on *F*^2^	Primary atom site location: structure-invariant direct methods
Least-squares matrix: full	Secondary atom site location: difference Fourier map
*R*[*F*^2^ > 2σ(*F*^2^)] = 0.051	Hydrogen site location: inferred from neighbouring sites
*wR*(*F*^2^) = 0.089	H-atom parameters constrained
*S* = 0.75	*w* = 1/[σ^2^(*F*_o_^2^) + (0.*P*)^2^] where *P* = (*F*_o_^2^ + 2*F*_c_^2^)/3
6988 reflections	(Δ/σ)_max_ < 0.001
435 parameters	Δρ_max_ = 0.35 e Å^−^^3^
12 restraints	Δρ_min_ = −0.24 e Å^−^^3^

### Special details

**Table:**

Geometry. All e.s.d.'s (except the e.s.d. in the dihedral angle between two l.s. planes) are estimated using the full covariance matrix. The cell e.s.d.'s are taken into account individually in the estimation of e.s.d.'s in distances, angles and torsion angles; correlations between e.s.d.'s in cell parameters are only used when they are defined by crystal symmetry. An approximate (isotropic) treatment of cell e.s.d.'s is used for estimating e.s.d.'s involving l.s. planes.
Refinement. Refinement of *F*^2^ against ALL reflections. The weighted *R*-factor *wR* and goodness of fit *S* are based on *F*^2^, conventional *R*-factors *R* are based on *F*, with *F* set to zero for negative *F*^2^. The threshold expression of *F*^2^ > σ(*F*^2^) is used only for calculating *R*-factors(gt) *etc*. and is not relevant to the choice of reflections for refinement. *R*-factors based on *F*^2^ are statistically about twice as large as those based on *F*, and *R*- factors based on ALL data will be even larger.

### Fractional atomic coordinates and isotropic or equivalent isotropic displacement parameters (Å^2^)

**Table:**

	*x*	*y*	*z*	*U*_iso_*/*U*_eq_	
Cu1	0.21585 (5)	0.98646 (5)	0.27053 (3)	0.04164 (17)	
P1	0.09334 (12)	0.97196 (11)	0.17417 (6)	0.0438 (3)	
N1	0.3161 (3)	0.8786 (3)	0.30319 (17)	0.0404 (9)	
N2	0.4998 (4)	0.8723 (4)	0.3817 (2)	0.0611 (11)	
N3	0.5058 (3)	1.0667 (3)	0.36417 (17)	0.0472 (9)	
N4	0.5552 (4)	1.2786 (4)	0.3743 (2)	0.0625 (11)	
N5	0.3128 (4)	1.1463 (3)	0.34393 (16)	0.0425 (9)	
Si1	0.21254 (13)	1.22563 (12)	0.39437 (6)	0.0481 (3)	
C1	0.2675 (4)	0.7442 (4)	0.2716 (2)	0.0368 (10)	
C2	0.3404 (5)	0.6878 (4)	0.2203 (2)	0.0436 (11)	
C3	0.2818 (5)	0.5576 (4)	0.1869 (2)	0.0602 (13)	
H3	0.3326	0.5185	0.1524	0.072*	
C4	0.1516 (6)	0.4838 (5)	0.2026 (3)	0.0649 (14)	
H4	0.1118	0.3947	0.1783	0.078*	
C5	0.0795 (5)	0.5377 (4)	0.2528 (3)	0.0602 (14)	
H5	−0.0098	0.4855	0.2641	0.072*	
C6	0.1343 (5)	0.6682 (4)	0.2878 (2)	0.0496 (12)	
C7	0.0520 (5)	0.7255 (5)	0.3453 (3)	0.0779 (17)	
H7	0.1096	0.8188	0.3657	0.093*	
C8	0.0293 (7)	0.6572 (7)	0.4055 (3)	0.148 (3)	
H8A	−0.0164	0.7020	0.4427	0.222*	
H8B	0.1228	0.6589	0.4250	0.222*	
H8C	−0.0331	0.5669	0.3873	0.222*	
C9	−0.0938 (6)	0.7191 (6)	0.3153 (3)	0.128 (2)	
H9A	−0.1489	0.6290	0.2913	0.192*	
H9B	−0.0806	0.7728	0.2814	0.192*	
H9C	−0.1464	0.7513	0.3536	0.192*	
C10	0.4816 (5)	0.7661 (4)	0.1990 (3)	0.0612 (13)	
H10	0.5233	0.8474	0.2375	0.073*	
C11	0.5904 (6)	0.6964 (5)	0.1891 (4)	0.153 (3)	
H11A	0.6046	0.6690	0.2319	0.230*	
H11B	0.6823	0.7545	0.1799	0.230*	
H11C	0.5555	0.6199	0.1489	0.230*	
C12	0.4540 (6)	0.8046 (6)	0.1327 (3)	0.137 (3)	
H12A	0.5463	0.8468	0.1161	0.205*	
H12B	0.3987	0.8650	0.1427	0.205*	
H12C	0.3989	0.7271	0.0961	0.205*	
C13	0.4329 (5)	0.9383 (4)	0.3489 (2)	0.0437 (11)	
C14	0.6398 (5)	0.9422 (5)	0.4222 (3)	0.106 (2)	
H14A	0.6997	1.0021	0.3968	0.159*	
H14B	0.6876	0.8801	0.4283	0.159*	
H14C	0.6260	0.9912	0.4685	0.159*	
C15	0.4316 (6)	0.7524 (5)	0.3966 (3)	0.110 (2)	
H15A	0.3271	0.7272	0.3851	0.164*	
H15B	0.4521	0.7599	0.4469	0.164*	
H15C	0.4681	0.6864	0.3683	0.164*	
C16	0.4503 (5)	1.1592 (4)	0.3590 (2)	0.0458 (11)	
C17	0.7011 (5)	1.3023 (4)	0.4024 (3)	0.0821 (17)	
H17A	0.7025	1.2352	0.4264	0.123*	
H17B	0.7363	1.3877	0.4362	0.123*	
H17C	0.7635	1.3002	0.3638	0.123*	
C18	0.5366 (5)	1.3833 (4)	0.3462 (3)	0.0809 (17)	
H18A	0.6148	1.4108	0.3169	0.121*	
H18B	0.5392	1.4569	0.3854	0.121*	
H18C	0.4436	1.3521	0.3176	0.121*	
C19	0.3128 (5)	1.3379 (4)	0.4780 (2)	0.0682 (14)	
H19A	0.3866	1.3053	0.4936	0.102*	
H19B	0.2455	1.3434	0.5143	0.102*	
H19C	0.3592	1.4242	0.4703	0.102*	
C20	0.0592 (4)	1.1020 (4)	0.4188 (2)	0.0745 (15)	
H20A	0.0047	1.0390	0.3759	0.112*	
H20B	−0.0043	1.1445	0.4453	0.112*	
H20C	0.0962	1.0571	0.4483	0.112*	
C21	0.1368 (5)	1.3199 (4)	0.3466 (2)	0.0824 (17)	
H21A	0.2157	1.3913	0.3371	0.124*	
H21B	0.0710	1.3551	0.3757	0.124*	
H21C	0.0839	1.2628	0.3018	0.124*	
C22	−0.0030 (4)	0.8179 (4)	0.1131 (2)	0.0485 (12)	
C23	0.0152 (5)	0.7052 (5)	0.1226 (3)	0.0733 (16)	
H23	0.0821	0.7099	0.1596	0.088*	
C24	−0.0622 (6)	0.5870 (5)	0.0792 (3)	0.103 (2)	
H24	−0.0520	0.5104	0.0881	0.124*	
C25	−0.1539 (6)	0.5771 (5)	0.0233 (3)	0.098 (2)	
H25	−0.2046	0.4949	−0.0074	0.117*	
C26	−0.1713 (6)	0.6880 (5)	0.0125 (3)	0.0842 (17)	
H26	−0.2359	0.6826	−0.0256	0.101*	
C27	−0.0970 (5)	0.8055 (5)	0.0559 (2)	0.0651 (14)	
H27	−0.1096	0.8813	0.0470	0.078*	
C28	−0.0413 (4)	1.0544 (4)	0.1788 (2)	0.0423 (11)	
C29	−0.1412 (5)	1.0359 (4)	0.2284 (2)	0.0566 (13)	
H29	−0.1351	0.9835	0.2593	0.068*	
C30	−0.2476 (5)	1.0912 (5)	0.2337 (3)	0.0728 (15)	
H30	−0.3143	1.0777	0.2681	0.087*	
C31	−0.2577 (5)	1.1664 (5)	0.1893 (3)	0.0782 (17)	
H31	−0.3317	1.2051	0.1927	0.094*	
C32	−0.1605 (6)	1.1860 (4)	0.1393 (3)	0.0721 (16)	
H32	−0.1671	1.2388	0.1087	0.087*	
C33	−0.0545 (5)	1.1292 (4)	0.1340 (2)	0.0505 (12)	
H33	0.0107	1.1415	0.0988	0.061*	
C34	0.2300 (4)	1.0584 (5)	0.1248 (2)	0.0475 (12)	
C35	0.3147 (5)	1.1830 (5)	0.1584 (2)	0.0566 (13)	
H35	0.2973	1.2218	0.2046	0.068*	
C36	0.4241 (5)	1.2518 (5)	0.1258 (3)	0.0685 (15)	
H36	0.4825	1.3370	0.1500	0.082*	
C37	0.4500 (6)	1.1987 (6)	0.0585 (3)	0.0771 (18)	
H37	0.5257	1.2464	0.0360	0.093*	
C38	0.3649 (6)	1.0763 (6)	0.0250 (3)	0.0772 (17)	
H38	0.3819	1.0386	−0.0215	0.093*	
C39	0.2543 (5)	1.0056 (5)	0.0570 (3)	0.0612 (14)	
H39	0.1953	0.9209	0.0323	0.073*	

### Atomic displacement parameters (Å^2^)

**Table:**

	*U*^11^	*U*^22^	*U*^33^	*U*^12^	*U*^13^	*U*^23^
Cu1	0.0472 (3)	0.0362 (3)	0.0419 (3)	0.0148 (3)	−0.0030 (3)	0.0093 (3)
P1	0.0514 (8)	0.0412 (7)	0.0408 (7)	0.0192 (6)	−0.0039 (6)	0.0088 (6)
N1	0.045 (2)	0.026 (2)	0.050 (2)	0.0119 (18)	−0.0095 (18)	0.0103 (19)
N2	0.058 (3)	0.049 (3)	0.078 (3)	0.020 (2)	−0.021 (2)	0.018 (2)
N3	0.039 (2)	0.041 (2)	0.055 (3)	0.011 (2)	−0.0054 (18)	0.004 (2)
N4	0.053 (3)	0.040 (3)	0.081 (3)	0.003 (2)	−0.006 (2)	0.009 (2)
N5	0.039 (2)	0.038 (2)	0.044 (2)	0.0099 (18)	−0.0058 (18)	0.0034 (19)
Si1	0.0529 (8)	0.0398 (8)	0.0497 (9)	0.0188 (7)	−0.0053 (7)	0.0034 (7)
C1	0.035 (3)	0.033 (3)	0.048 (3)	0.013 (2)	−0.004 (2)	0.016 (2)
C2	0.045 (3)	0.042 (3)	0.048 (3)	0.019 (2)	0.001 (2)	0.013 (3)
C3	0.071 (4)	0.045 (3)	0.066 (4)	0.026 (3)	0.005 (3)	0.008 (3)
C4	0.081 (4)	0.036 (3)	0.076 (4)	0.022 (3)	−0.001 (3)	0.009 (3)
C5	0.057 (3)	0.041 (3)	0.083 (4)	0.009 (3)	0.006 (3)	0.025 (3)
C6	0.047 (3)	0.043 (3)	0.064 (3)	0.019 (3)	0.001 (3)	0.017 (3)
C7	0.064 (4)	0.049 (3)	0.126 (5)	0.019 (3)	0.036 (4)	0.031 (4)
C8	0.187 (7)	0.198 (8)	0.106 (6)	0.102 (6)	0.073 (5)	0.069 (6)
C9	0.093 (5)	0.145 (6)	0.187 (7)	0.072 (5)	0.050 (5)	0.072 (6)
C10	0.058 (3)	0.058 (3)	0.071 (4)	0.021 (3)	0.014 (3)	0.018 (3)
C11	0.076 (5)	0.116 (6)	0.289 (10)	0.050 (4)	0.067 (5)	0.066 (6)
C12	0.118 (5)	0.187 (7)	0.098 (5)	0.003 (5)	0.015 (4)	0.085 (5)
C13	0.042 (3)	0.046 (3)	0.046 (3)	0.019 (3)	0.009 (2)	0.011 (3)
C14	0.082 (4)	0.087 (5)	0.149 (6)	0.030 (4)	−0.054 (4)	0.029 (4)
C15	0.120 (5)	0.078 (4)	0.122 (5)	0.006 (4)	−0.054 (4)	0.046 (4)
C16	0.052 (3)	0.038 (3)	0.037 (3)	0.005 (3)	0.007 (2)	0.003 (2)
C17	0.053 (3)	0.062 (4)	0.101 (4)	−0.008 (3)	−0.012 (3)	0.001 (3)
C18	0.093 (4)	0.045 (3)	0.085 (4)	−0.005 (3)	0.003 (3)	0.017 (3)
C19	0.078 (4)	0.064 (3)	0.058 (3)	0.028 (3)	0.000 (3)	0.002 (3)
C20	0.060 (3)	0.075 (4)	0.078 (4)	0.017 (3)	0.011 (3)	0.006 (3)
C21	0.099 (4)	0.064 (4)	0.093 (4)	0.047 (3)	−0.019 (3)	0.011 (3)
C22	0.058 (3)	0.048 (3)	0.041 (3)	0.024 (3)	−0.010 (2)	0.006 (2)
C23	0.096 (4)	0.051 (3)	0.073 (4)	0.030 (3)	−0.031 (3)	0.009 (3)
C24	0.151 (6)	0.046 (4)	0.100 (5)	0.035 (4)	−0.059 (4)	−0.004 (3)
C25	0.122 (5)	0.052 (4)	0.097 (5)	0.025 (4)	−0.055 (4)	−0.014 (4)
C26	0.100 (4)	0.069 (4)	0.076 (4)	0.039 (4)	−0.042 (3)	−0.008 (3)
C27	0.082 (4)	0.052 (3)	0.057 (3)	0.030 (3)	−0.023 (3)	−0.001 (3)
C28	0.043 (3)	0.036 (3)	0.047 (3)	0.014 (2)	−0.004 (2)	0.006 (2)
C29	0.054 (3)	0.057 (3)	0.061 (3)	0.022 (3)	0.004 (3)	0.014 (3)
C30	0.058 (4)	0.074 (4)	0.086 (4)	0.028 (3)	0.018 (3)	0.010 (4)
C31	0.057 (4)	0.064 (4)	0.114 (5)	0.035 (3)	−0.008 (4)	0.004 (4)
C32	0.070 (4)	0.062 (4)	0.095 (4)	0.033 (3)	−0.010 (3)	0.024 (3)
C33	0.057 (3)	0.046 (3)	0.053 (3)	0.025 (3)	0.000 (2)	0.012 (3)
C34	0.046 (3)	0.062 (3)	0.047 (3)	0.033 (3)	0.002 (2)	0.018 (3)
C35	0.050 (3)	0.062 (4)	0.053 (3)	0.015 (3)	0.003 (3)	0.010 (3)
C36	0.052 (3)	0.070 (4)	0.085 (4)	0.013 (3)	0.000 (3)	0.033 (4)
C37	0.067 (4)	0.101 (5)	0.084 (5)	0.034 (4)	0.020 (4)	0.053 (4)
C38	0.083 (4)	0.114 (5)	0.055 (4)	0.051 (4)	0.024 (3)	0.032 (4)
C39	0.073 (4)	0.068 (4)	0.054 (3)	0.036 (3)	0.008 (3)	0.017 (3)

### Geometric parameters (Å, º)

**Table:**

Cu1—N5	1.964 (3)	C14—H14C	0.9800
Cu1—N1	1.980 (3)	C15—H15A	0.9800
Cu1—P1	2.1651 (12)	C15—H15B	0.9800
P1—C22	1.811 (4)	C15—H15C	0.9800
P1—C28	1.824 (4)	C17—H17A	0.9800
P1—C34	1.824 (4)	C17—H17B	0.9800
N1—C13	1.328 (5)	C17—H17C	0.9800
N1—C1	1.409 (4)	C18—H18A	0.9800
N2—C13	1.379 (5)	C18—H18B	0.9800
N2—C15	1.403 (5)	C18—H18C	0.9800
N2—C14	1.457 (5)	C19—H19A	0.9800
N3—C16	1.335 (5)	C19—H19B	0.9800
N3—C13	1.349 (5)	C19—H19C	0.9800
N4—C16	1.371 (5)	C20—H20A	0.9800
N4—C17	1.444 (5)	C20—H20B	0.9800
N4—C18	1.464 (5)	C20—H20C	0.9800
N5—C16	1.329 (5)	C21—H21A	0.9800
N5—Si1	1.702 (3)	C21—H21B	0.9800
Si1—C19	1.853 (4)	C21—H21C	0.9800
Si1—C20	1.854 (4)	C22—C23	1.384 (5)
Si1—C21	1.865 (4)	C22—C27	1.390 (5)
C1—C2	1.387 (5)	C23—C24	1.369 (6)
C1—C6	1.404 (5)	C23—H23	0.9500
C2—C3	1.379 (5)	C24—C25	1.365 (6)
C2—C10	1.512 (5)	C24—H24	0.9500
C3—C4	1.370 (6)	C25—C26	1.369 (6)
C3—H3	0.9500	C25—H25	0.9500
C4—C5	1.356 (6)	C26—C27	1.357 (6)
C4—H4	0.9500	C26—H26	0.9500
C5—C6	1.386 (5)	C27—H27	0.9500
C5—H5	0.9500	C28—C33	1.380 (5)
C6—C7	1.531 (6)	C28—C29	1.392 (5)
C7—C9	1.510 (6)	C29—C30	1.363 (5)
C7—C8	1.533 (6)	C29—H29	0.9500
C7—H7	1.0000	C30—C31	1.368 (6)
C8—H8A	0.9800	C30—H30	0.9500
C8—H8B	0.9800	C31—C32	1.382 (6)
C8—H8C	0.9800	C31—H31	0.9500
C9—H9A	0.9800	C32—C33	1.371 (5)
C9—H9B	0.9800	C32—H32	0.9500
C9—H9C	0.9800	C33—H33	0.9500
C10—C11	1.497 (6)	C34—C35	1.378 (5)
C10—C12	1.506 (6)	C34—C39	1.378 (5)
C10—H10	1.0000	C35—C36	1.372 (5)
C11—H11A	0.9800	C35—H35	0.9500
C11—H11B	0.9800	C36—C37	1.374 (6)
C11—H11C	0.9800	C36—H36	0.9500
C12—H12A	0.9800	C37—C38	1.362 (6)
C12—H12B	0.9800	C37—H37	0.9500
C12—H12C	0.9800	C38—C39	1.382 (6)
C14—H14A	0.9800	C38—H38	0.9500
C14—H14B	0.9800	C39—H39	0.9500
			
N5—Cu1—N1	95.88 (14)	N2—C15—H15B	109.5
N5—Cu1—P1	126.26 (10)	H15A—C15—H15B	109.5
N1—Cu1—P1	135.59 (11)	N2—C15—H15C	109.5
C22—P1—C28	100.94 (18)	H15A—C15—H15C	109.5
C22—P1—C34	105.5 (2)	H15B—C15—H15C	109.5
C28—P1—C34	103.48 (19)	N5—C16—N3	127.8 (4)
C22—P1—Cu1	122.58 (14)	N5—C16—N4	121.1 (4)
C28—P1—Cu1	119.03 (14)	N3—C16—N4	111.1 (4)
C34—P1—Cu1	103.22 (14)	N4—C17—H17A	109.5
C13—N1—C1	123.6 (3)	N4—C17—H17B	109.5
C13—N1—Cu1	118.2 (3)	H17A—C17—H17B	109.5
C1—N1—Cu1	118.0 (2)	N4—C17—H17C	109.5
C13—N2—C15	125.5 (4)	H17A—C17—H17C	109.5
C13—N2—C14	119.3 (4)	H17B—C17—H17C	109.5
C15—N2—C14	112.9 (4)	N4—C18—H18A	109.5
C16—N3—C13	125.8 (4)	N4—C18—H18B	109.5
C16—N4—C17	123.6 (4)	H18A—C18—H18B	109.5
C16—N4—C18	120.5 (4)	N4—C18—H18C	109.5
C17—N4—C18	114.1 (4)	H18A—C18—H18C	109.5
C16—N5—Si1	126.4 (3)	H18B—C18—H18C	109.5
C16—N5—Cu1	111.5 (3)	Si1—C19—H19A	109.5
Si1—N5—Cu1	119.71 (19)	Si1—C19—H19B	109.5
N5—Si1—C19	113.54 (19)	H19A—C19—H19B	109.5
N5—Si1—C20	107.65 (19)	Si1—C19—H19C	109.5
C19—Si1—C20	106.9 (2)	H19A—C19—H19C	109.5
N5—Si1—C21	112.7 (2)	H19B—C19—H19C	109.5
C19—Si1—C21	107.8 (2)	Si1—C20—H20A	109.5
C20—Si1—C21	107.9 (2)	Si1—C20—H20B	109.5
C2—C1—C6	119.4 (4)	H20A—C20—H20B	109.5
C2—C1—N1	121.7 (4)	Si1—C20—H20C	109.5
C6—C1—N1	118.6 (4)	H20A—C20—H20C	109.5
C3—C2—C1	119.4 (4)	H20B—C20—H20C	109.5
C3—C2—C10	119.0 (4)	Si1—C21—H21A	109.5
C1—C2—C10	121.6 (4)	Si1—C21—H21B	109.5
C4—C3—C2	121.1 (5)	H21A—C21—H21B	109.5
C4—C3—H3	119.4	Si1—C21—H21C	109.5
C2—C3—H3	119.4	H21A—C21—H21C	109.5
C5—C4—C3	120.0 (5)	H21B—C21—H21C	109.5
C5—C4—H4	120.0	C23—C22—C27	117.0 (4)
C3—C4—H4	120.0	C23—C22—P1	119.7 (3)
C4—C5—C6	120.8 (5)	C27—C22—P1	123.3 (4)
C4—C5—H5	119.6	C24—C23—C22	120.6 (4)
C6—C5—H5	119.6	C24—C23—H23	119.7
C5—C6—C1	119.3 (4)	C22—C23—H23	119.7
C5—C6—C7	119.6 (4)	C25—C24—C23	121.3 (5)
C1—C6—C7	121.1 (4)	C25—C24—H24	119.4
C9—C7—C6	111.2 (5)	C23—C24—H24	119.4
C9—C7—C8	108.4 (5)	C24—C25—C26	118.7 (5)
C6—C7—C8	112.8 (4)	C24—C25—H25	120.6
C9—C7—H7	108.1	C26—C25—H25	120.6
C6—C7—H7	108.1	C27—C26—C25	120.5 (5)
C8—C7—H7	108.1	C27—C26—H26	119.7
C7—C8—H8A	109.5	C25—C26—H26	119.7
C7—C8—H8B	109.5	C26—C27—C22	121.8 (4)
H8A—C8—H8B	109.5	C26—C27—H27	119.1
C7—C8—H8C	109.5	C22—C27—H27	119.1
H8A—C8—H8C	109.5	C33—C28—C29	117.8 (4)
H8B—C8—H8C	109.5	C33—C28—P1	123.9 (4)
C7—C9—H9A	109.5	C29—C28—P1	118.2 (3)
C7—C9—H9B	109.5	C30—C29—C28	121.6 (4)
H9A—C9—H9B	109.5	C30—C29—H29	119.2
C7—C9—H9C	109.5	C28—C29—H29	119.2
H9A—C9—H9C	109.5	C29—C30—C31	119.7 (5)
H9B—C9—H9C	109.5	C29—C30—H30	120.2
C11—C10—C12	110.3 (5)	C31—C30—H30	120.2
C11—C10—C2	113.1 (4)	C30—C31—C32	120.1 (5)
C12—C10—C2	109.7 (4)	C30—C31—H31	120.0
C11—C10—H10	107.9	C32—C31—H31	120.0
C12—C10—H10	107.9	C33—C32—C31	119.9 (5)
C2—C10—H10	107.9	C33—C32—H32	120.1
C10—C11—H11A	109.5	C31—C32—H32	120.1
C10—C11—H11B	109.5	C32—C33—C28	121.0 (4)
H11A—C11—H11B	109.5	C32—C33—H33	119.5
C10—C11—H11C	109.5	C28—C33—H33	119.5
H11A—C11—H11C	109.5	C35—C34—C39	118.6 (4)
H11B—C11—H11C	109.5	C35—C34—P1	117.5 (4)
C10—C12—H12A	109.5	C39—C34—P1	123.9 (4)
C10—C12—H12B	109.5	C36—C35—C34	120.8 (5)
H12A—C12—H12B	109.5	C36—C35—H35	119.6
C10—C12—H12C	109.5	C34—C35—H35	119.6
H12A—C12—H12C	109.5	C35—C36—C37	120.8 (5)
H12B—C12—H12C	109.5	C35—C36—H36	119.6
N1—C13—N3	124.6 (4)	C37—C36—H36	119.6
N1—C13—N2	122.7 (4)	C38—C37—C36	118.5 (5)
N3—C13—N2	112.5 (4)	C38—C37—H37	120.8
N2—C14—H14A	109.5	C36—C37—H37	120.8
N2—C14—H14B	109.5	C37—C38—C39	121.6 (5)
H14A—C14—H14B	109.5	C37—C38—H38	119.2
N2—C14—H14C	109.5	C39—C38—H38	119.2
H14A—C14—H14C	109.5	C34—C39—C38	119.8 (5)
H14B—C14—H14C	109.5	C34—C39—H39	120.1
N2—C15—H15A	109.5	C38—C39—H39	120.1
